# Role of drug-dependent transporter modulation on the chemosensitivity of cholangiocarcinoma

**DOI:** 10.18632/oncotarget.21624

**Published:** 2017-10-06

**Authors:** Nerea Urtasun, Clara Boces-Pascual, Loreto Boix, Jordi Bruix, Marçal Pastor-Anglada, Sandra Pérez-Torras

**Affiliations:** ^1^ Molecular Pharmacology and Experimental Therapeutics (MPET), Section Biochemistry and Molecular Pharmacology, Department of Biochemistry and Molecular Biomedicine, Institute of Biomedicine (IBUB), University of Barcelona, Barcelona, Spain; ^2^ Oncology Program, CIBER ehd, National Biomedical Research Institute on Liver and Gastrointestinal Diseases, Instituto de Salud Carlos III, Barcelona, Spain; ^3^ Barcelona Clinic Liver Cancer (BCLC) Group, Liver Unit, Hospital Clínic of Barcelona, University of Barcelona, Institut d’Investigacions Biomèdiques August Pi i Sunyer (IDIBAPS), Fundació Clínic per a la Recerca Biomèdica (FCRB), Barcelona, Spain

**Keywords:** transporter, cholangiocarcinoma, gemcitabine, cisplatin, chemoresistance

## Abstract

Cholangiocarcinoma (CCA) is a heterogeneous group of malignancies with limited therapeutic options. Curative therapy is limited to surgery whereas chemotherapy treatments are the election option for unresectable or metastatic cholangiocarcinoma. Cisplatin plus gemcitabine is the reference chemotherapy regimen, albeit the contribution to the median overall survival barely reaches one year. Drug transporters are undoubtedly a limiting step for drug bioavailability and have been clearly related to chemoresistance. Several members of the SoLute Carrier (SLC) superfamily involved in the uptake of anticancer drugs used to treat cholangiocarcinoma are downregulated in these tumors. This study shows the increase in the expression of specific drug transporters exerted by cisplatin treatment thereby enhancing their transport activity. Combination treatments of cisplatin with selected drugs as gemcitabine and sorafenib take in by these transporters at the desired combination schedule induced synergy. These data support the concept that proper administration pattern could favor treatment outcome.

## INTRODUCTION

Cholangiocarcinoma (CCA) is a heterogeneous group of malignancies, which have in common features of biliary tract differentiation. Efforts to characterize and classify the different subtypes of this cancer at the histological and molecular level are being carried out to eventually implement a personalized treatment [1–3]. CCA is a marked chemoresistant malignancy, for which surgery represents the only curative treatment, and systemic chemotherapy remains the mainstay palliative treatment modality for unresectable or metastatic disease. Gemcitabine combined with cisplatin is the most effective treatment option for locally advanced or metastatic CCA. Combination of these drugs improves progression-free and overall survival, although reaching almost a mere one year of median overall survival [4]. The cisplatin combined with gemcitabine regimen also represents a cost-effective alternative treatment compared with gemcitabine monotherapy [5].

Drug bioavailability and responsiveness may indeed depend upon the so-called transportome profile of the tumor and its associated pharmacogenetics, thereby providing novel networks and genes within them likely to contribute to chemoresistance [6–8]. Mechanisms of chemoresistance to cholangiocarcinoma current treatments have been identified and classified based upon the steps implicated in drug transport, metabolism, and action [9, 10]. Indeed, drug transporters encompass the first limiting step, involving several members of the SLC superfamily in mediating drug influx through the cell membrane. Uptake of nucleoside analogs such as gemcitabine and 5-fluorouracil mainly relies on Nucleoside Transporters (NTs), both the Equilibrative Nucleoside Transporters (ENTs) and the Concentrative Nucleoside Transporters (CNTs), to enter the cell [7]. Cationic drugs like platinum derivatives and tyrosine kinase inhibitors as cisplatin and sorafenib, respectively, are taken up in part through Organic Cation Transporters (OCTs) [11, 12]. Moreover, sorafenib uptake is also mediated by Organic Anion Transporting Polypeptides (OATPs) [13]. Unfortunately, most of these drug transporters are downregulated in cholangiocarcinoma, thereby limiting the response to these treatments [14–17].

Therefore, in this study we addressed the effect of cisplatin treatment to modulate drug transporters to attempt a chemosensitization strategy based on the temporal drug administration pattern.

## RESULTS

### Cholangiocarcinoma drug transporters

**Figure 1:**
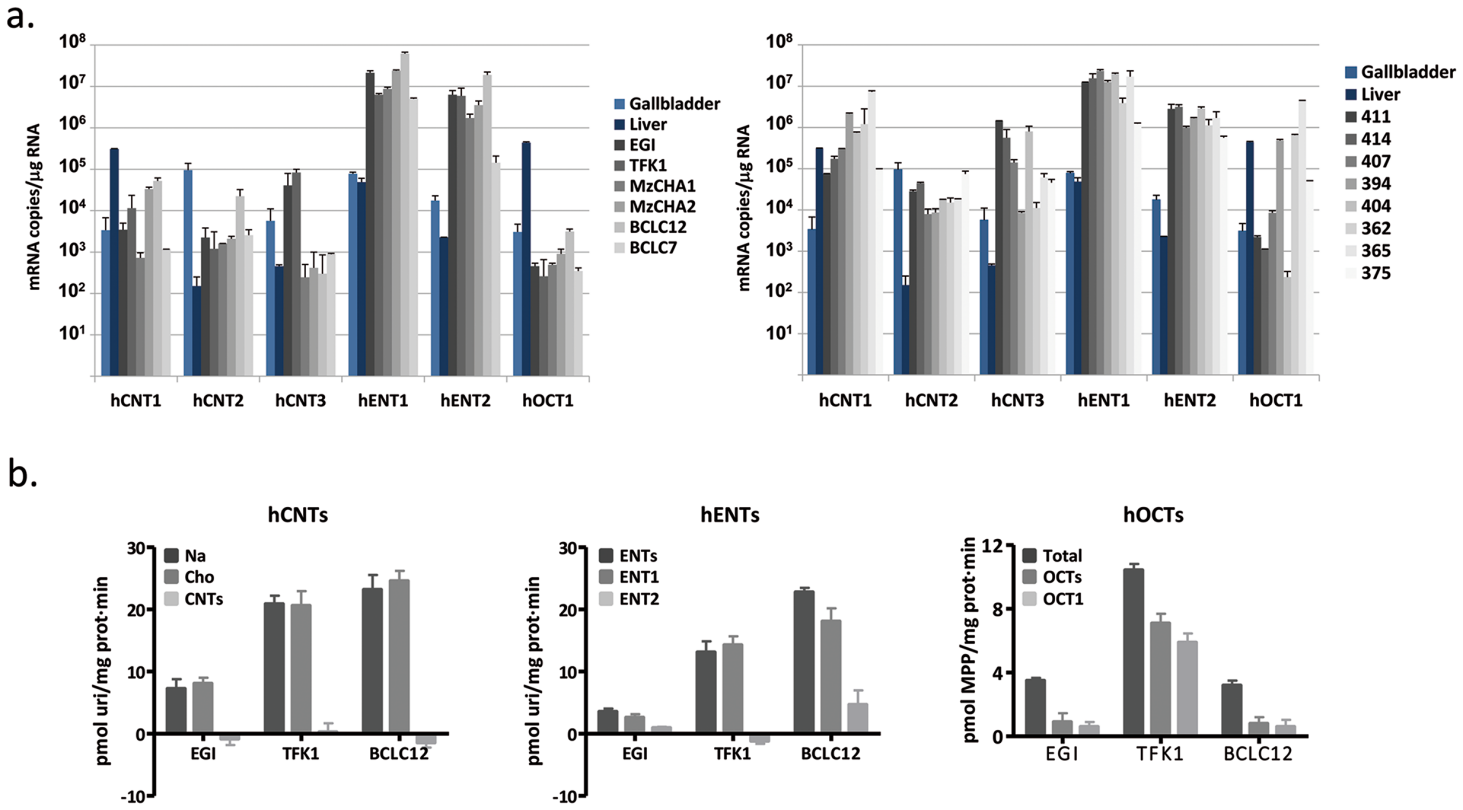
Cholangiocarcinoma drug transporters characterization **(A)** mRNA expression of hNTs and hOCT1 in 6 cholangiocarcinoma derived cell lines (left) and 8 tumor samples (right). Four gallbladder (light blue) and one liver (dark blue) tissues were assessed as a reference. **(B)** Transport activity was determined in EGI-1, TFK-1 and BCLC12 cell lines. hCNTs (left) sodium-dependent uptake of [^3^H]uridine (light gray) was determined as uptake in NaCl medium (black) minus uptake in choline chloride (gray). hENTs (middle) uptake of [^3^H]uridine was determined as total uptake in choline chloride (black) minus uptake inhibited by 1μM NBTI for the hENT1 (gray) or 10μM dipyridamole for the hENT2 (light gray). hOCTs (right) uptake of [^3^H]MPP was determined as total uptake (black) minus uptake inhibited by 100μM d22 (gray) or 100μM quinidine (light gray) for the hOCT1. Results are mean ± S.E.M. (n=3).

NTs and OCT1 expression from tumor samples (Supplementary Table 1) and cholangiocarcinoma derived cell lines were analyzed in order to determine the transporter profile of the currently used drugs to treat this malignancy. Total mRNA copies from 4 gallbladder samples and a commercially available sample of healthy liver tissue (Ambion, Thermo Fisher Scientific Inc.) were also analyzed as a reference. Tumor samples showed a general increase in hENT1 and hENT2 expression likewise cell lines, whereas hCNTs and hOCT1 expression exhibited a more variable pattern (Figure 1A). These results prompted us to increase the number of samples and look for appropriated controls. The analysis of public data (GEO:GSE26566,[1]) from 59 paired samples of CCA tumors and its surrounding liver showed a significant decrease of hCNT1 and hOCT1 expression in tumors, whereas hCNT3 was increased (Supplementary Figure 1). However, when drug transporters expression was compared between cholangiocarcinoma and 6 normal intrahepatic bile duct samples obtained from the same cohort, a trend to diminish for almost all the high affinity nucleoside transporters and hOCT1 was observed with no general tendency to increase even considering the few normal intrahepatic bile duct samples.

Three different cholangiocarcinoma cell lines were chosen to determine their uptake features, one derived from an intrahepatic malignancy (BCLC12) and the other two from extrahepatic tumors (TFK-1 and EGI-1) (Figure 1B). All of them relied on hENTs to grant nucleosides uptake and mainly in hENT1, although BCLC12 also showed hENT2 activity. However, none of them displayed nucleoside uptake depending on hCNTs, despite the mRNA expression of all family members. The three cell lines exhibited hOCTs uptake at different levels and specifically hOCT1, being TFK-1 the cell line with the highest transport activity.

### Cisplatin treatment enhances drug transporters activity

**Figure 2:**
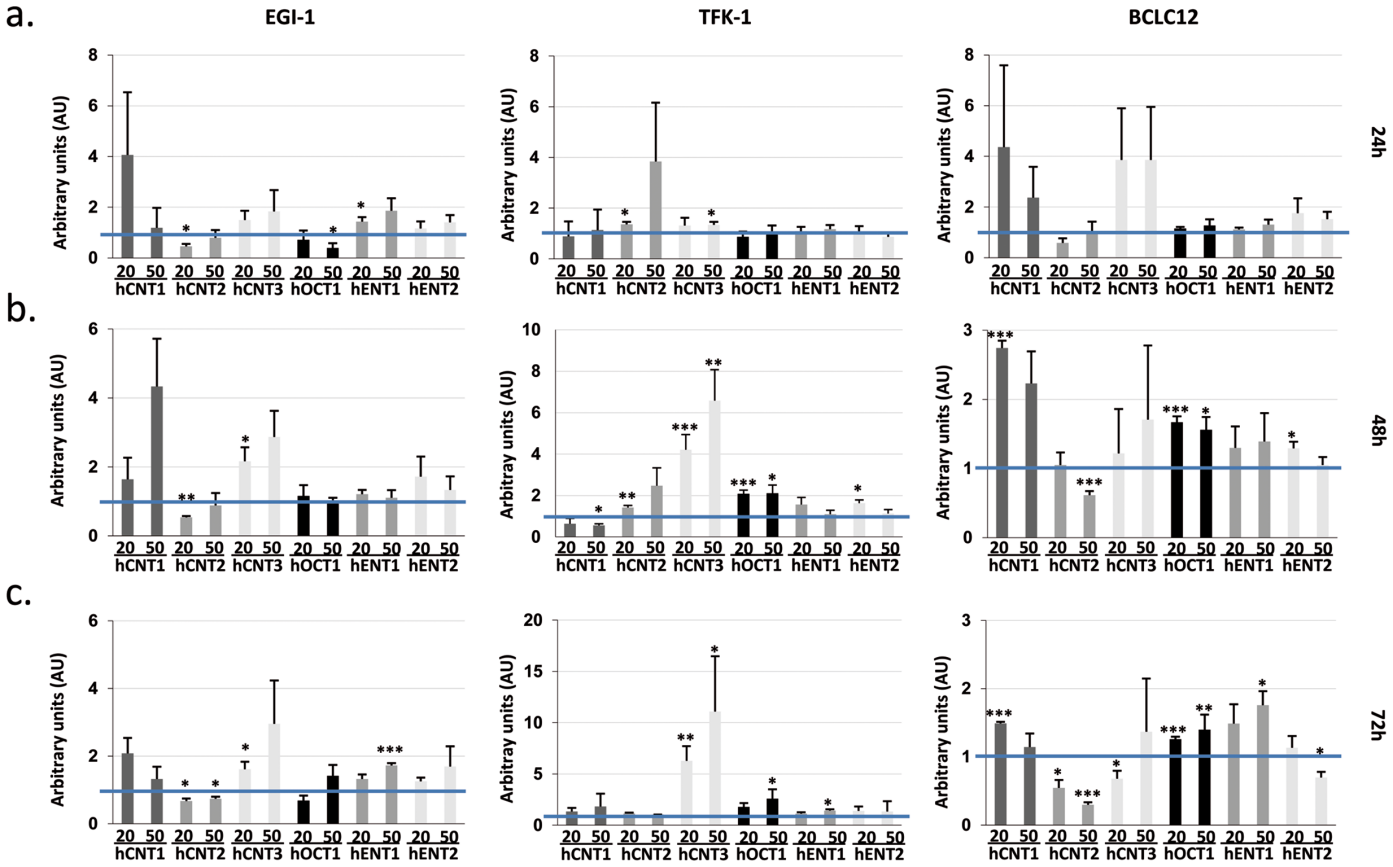
Cisplatin enhances drug transporter expression EGI-1, TFK-1 and BCLC12 cell lines were treated with their corresponding IC20 (20) or IC50 (50) cisplatin dose for 24h **(A)**, 48h **(B)** and 72h **(C)**. mRNA expression levels of hCNTs, hOCT1 and hENTs were determined by RT-PCR. Results are mean ± S.E.M. (n=3-4). Statistical significance was determined with Student's t-test; p<0.05^*^, p<0.01^**^, p<0.005^***^.

**Figure 3:**
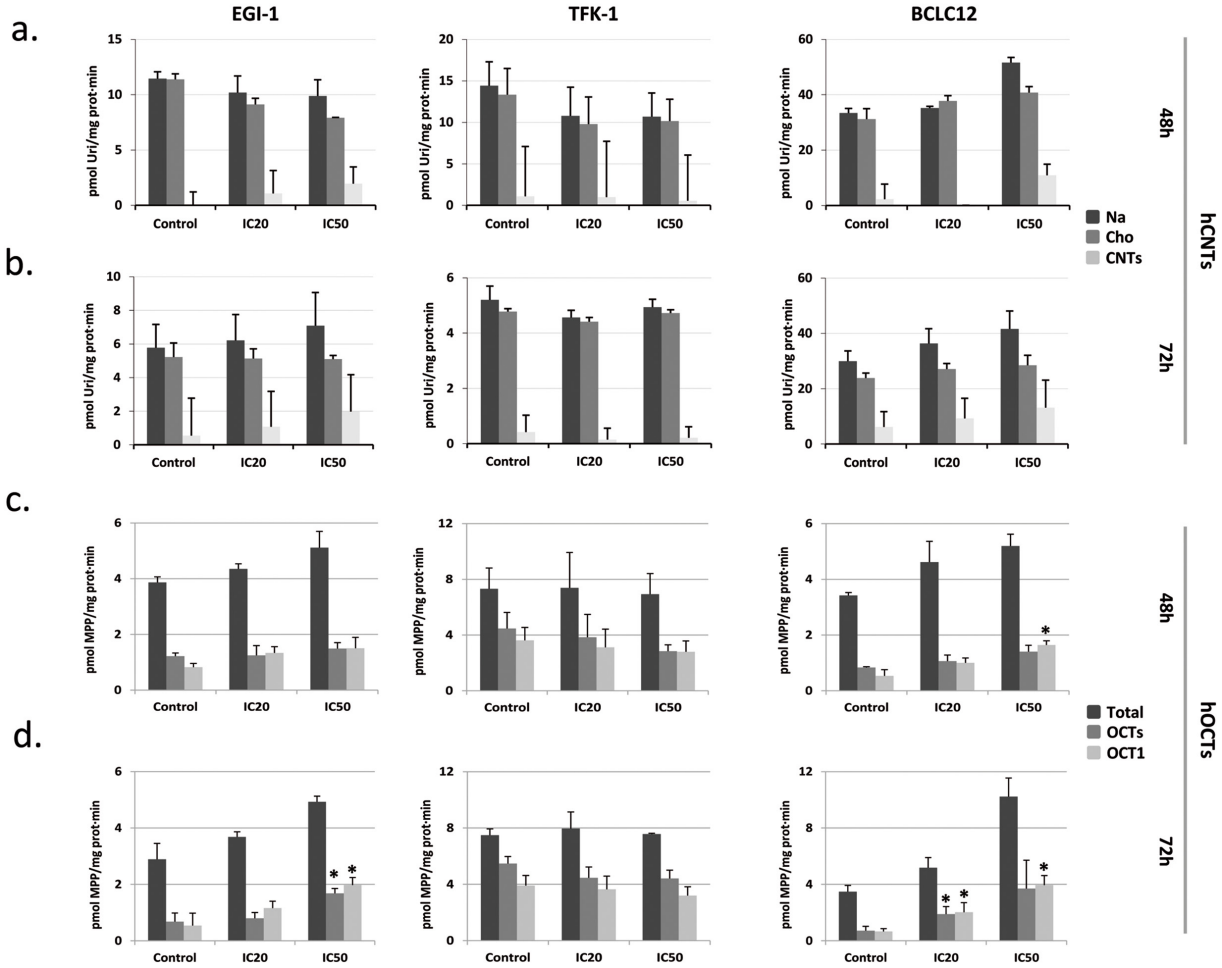
Cisplatin modulates hCNTs and hOCTs transport activity EGI-1, TFK-1 and BCLC12 cell lines were treated with their corresponding IC20 (20) or IC50 (50) cisplatin dose for 48h **(A, C)** and 72h **(B, D)**. hCNTs sodium-dependent uptake of [^3^H]uridine (light gray) was determined as uptake in NaCl medium (black) minus uptake in choline chloride (gray) (A, B). hOCTs uptake of [^3^H]MPP was determined as total uptake (black) minus uptake inhibited by d22 (gray) or quinidine (light gray) for the hOCT1 (C, D). Results are expressed as mean ± S.E.M. (n=2-3). Statistical significance was determined with Student's t-test; p<0.05^*^, p<0.01^**^, p<0.005^***^.

Previous works showed alterations in the mRNA levels of some members of the SLC gene superfamily in liver cancer cell lines after 72h of cisplatin treatment at IC50 dose [18]. These prompted us to analyze the expression of several drug transporters after cisplatin treatment in cholangiocarcinoma. Cell lines were treated with their corresponding IC20 or IC50 dose of cisplatin (Supplementary Figure 2) and hCNTs, hOCT1, hENT1 and hENT2 expression was determined 24, 48 and 72h later (Figure 2). Increases in the drug transporters hCNT1, hCNT3, hOCT1 and hENT1 were observed in the three cell lines especially from 48h treatment, although with variable extent depending on cell line. Analysis of nucleoside transport under the same conditions, showed a trend to increase in the hCNT-related sodium-dependent nucleoside uptake in EGI-1 and BCLC12 cell lines at 48h and 72h after treatment (Figure 3). Moreover, both cell lines displayed a significant increase in hOCTs-dependent uptake and in particular showed a significant increment in hOCT1 uptake at 72h after the IC50 cisplatin treatment (Figure 3). However, despite the significant increase observed in hCNT3 and hOCT1 at 48h, no activity changes were observed in the TFK-1 cell line for any of the analyzed transporters.

### Proper temporal administration pattern improves drug cytotoxic effect

**Figure 4:**
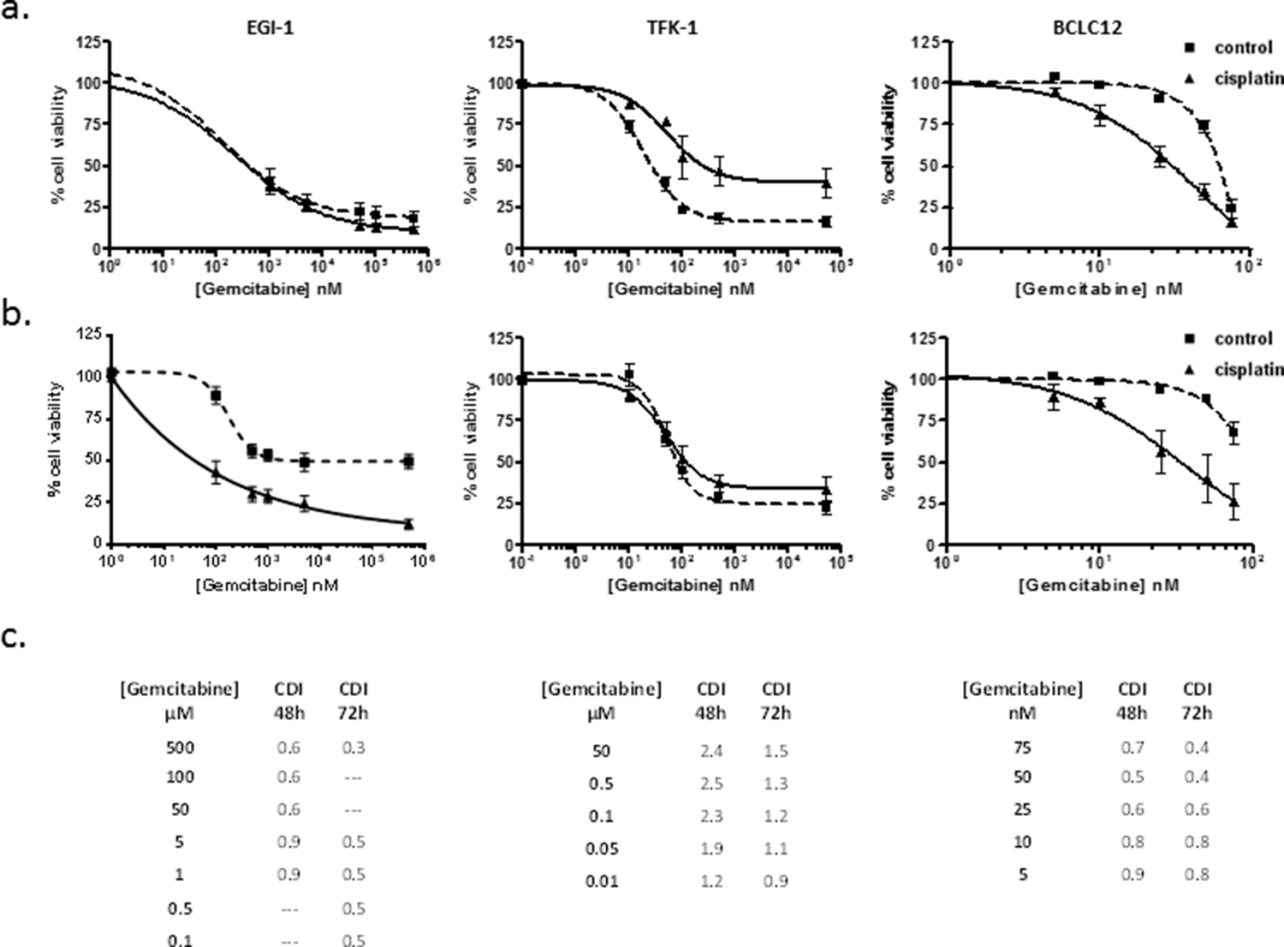
Gemcitabine cytotoxic effect improves with the administration temporal pattern Dose-response curves in EGI-1, TFK-1 and BCLC12 cell lines combining cisplatin and gemcitabine. Cells were treated with IC20 cisplatin dose (solid line) or vehicle (dashed line) and 48h **(A)** and 72h **(B)** later were treated with gemcitabine increasing doses. **(C)** CDI values for CDDP and gemcitabine combination treatments at 48h and 72h. Results are expressed as mean ± S.E.M. (n=3).

Currently used drugs to treat cholangiocarcinoma comprise nucleoside analogs such as gemcitabine and 5-fluorouracil in combination with cisplatin. The raise in NTs expression and activity after cisplatin treatment suggests that the drug administration temporal pattern has to be taken into account in order to increase drug-induced cytotoxicity. Since hCNT1, hCNT3 and hENT1 are able to efficiently transport gemcitabine, combination treatment was addressed at the time points where the increment was detected, compared to co-administration (Supplementary Figure 3). Dose-response curves to gemcitabine were performed 48 and 72 hours after cisplatin treatment with the corresponding IC20 dose. Interestingly, the cell lines EGI-1 and BCLC12, which showed an increase in hCNTs-dependent transport activity, displayed a synergy in the combination treatment with the highest effect observed at 72h (Figure 4, Supplementary Figure 4 24h). On the contrary, the TFK-1 cell line exhibited no synergy, which is consistent with the lack of increase in transport activity at these conditions. Furthermore, combination treatment with 5-fluorouracil, which is internalized by the NTs hENT1 and hENT2, and hOAT2, displayed similar results as obtained with gemcitabine combination in the three cell lines, showing only synergy in EGI-1 and BCLC12 (Supplementary Figure 5).

**Figure 5:**
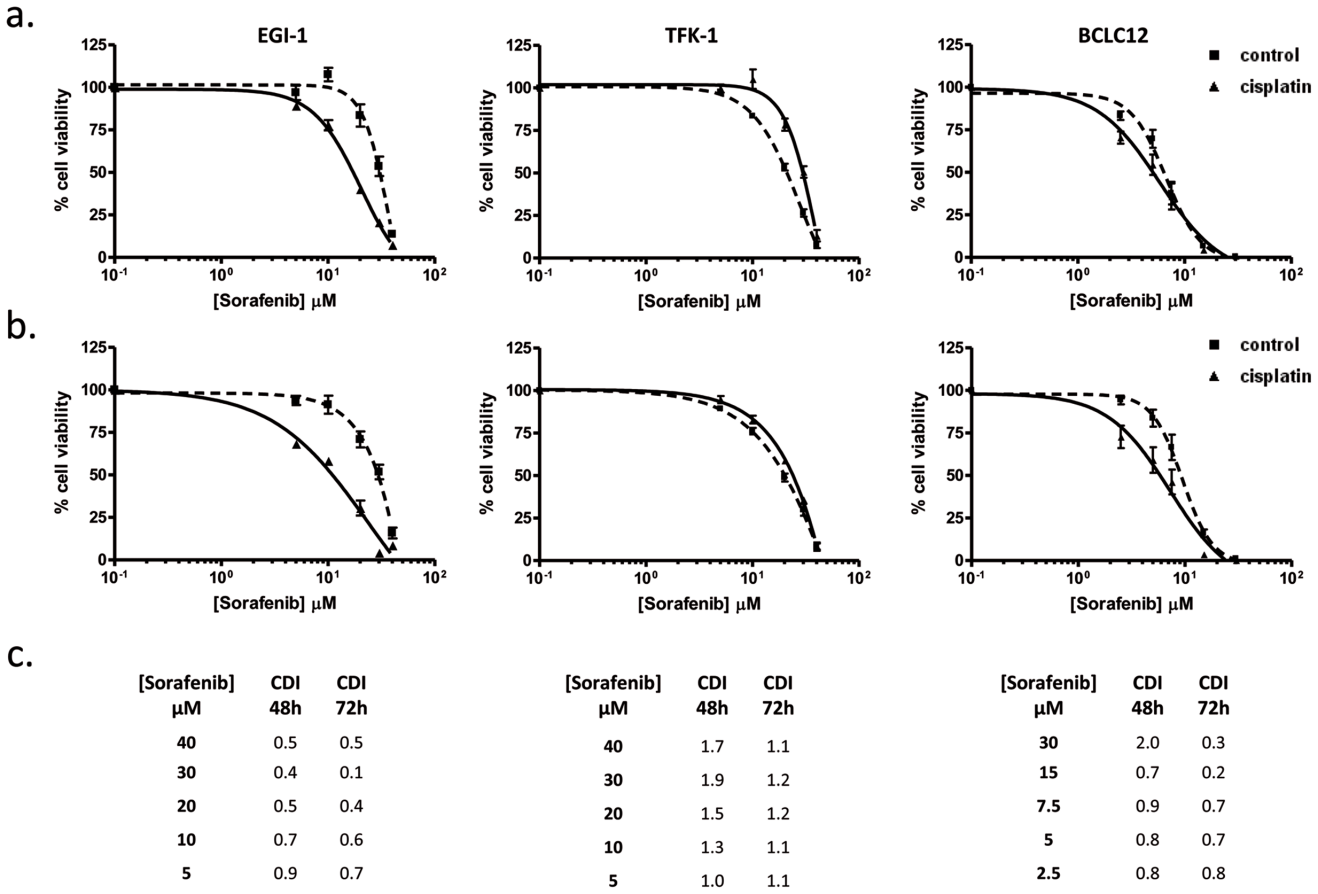
Sorafenib cytotoxic effect improves with the administration temporal pattern Dose-response curves in EGI-1, TFK-1 and BCLC12 cell lines combining cisplatin and sorafenib. Cells were treated with IC20 cisplatin dose (solid line) or vehicle (dashed line) and 48h **(A)** and 72h **(B)** later were treated with sorafenib increasing doses. **(C)** CDI values for CDDP and sorafenib combination treatments at 48h and 72h. Results are expressed as mean ± S.E.M. (n=3).

Following the same evidence, the increase observed in hOCT1-dependent transport prompted us to explore additional combination treatments. Thus, the hOCT1-transported drug sorafenib was combined following the pattern previously established. Cell lines were treated with cisplatin IC20 dose and after 48 and 72 hours, cells were incubated with increasing sorafenib doses. EGI-1 and BCLC12 exhibited higher sensitivity to sorafenib treatment after cisplatin administration (Figure 5) compared to co-administration pattern (Supplementary Figure 6). However, TFK-1 cells did not showed any improvement but even a trend to antagonism (Figure 5 and Supplementary Figure 6). Again, the synergy observed in EGI-1 and BCLC12 cell lines correlated with the increment displayed in hOCT1-dependent uptake (Figure 3). Furthermore, similar results were observed combining cisplatin with lower doses of paclitaxel, which is also transported by OCT1 (Supplementary Figure 7).

**Figure 6:**
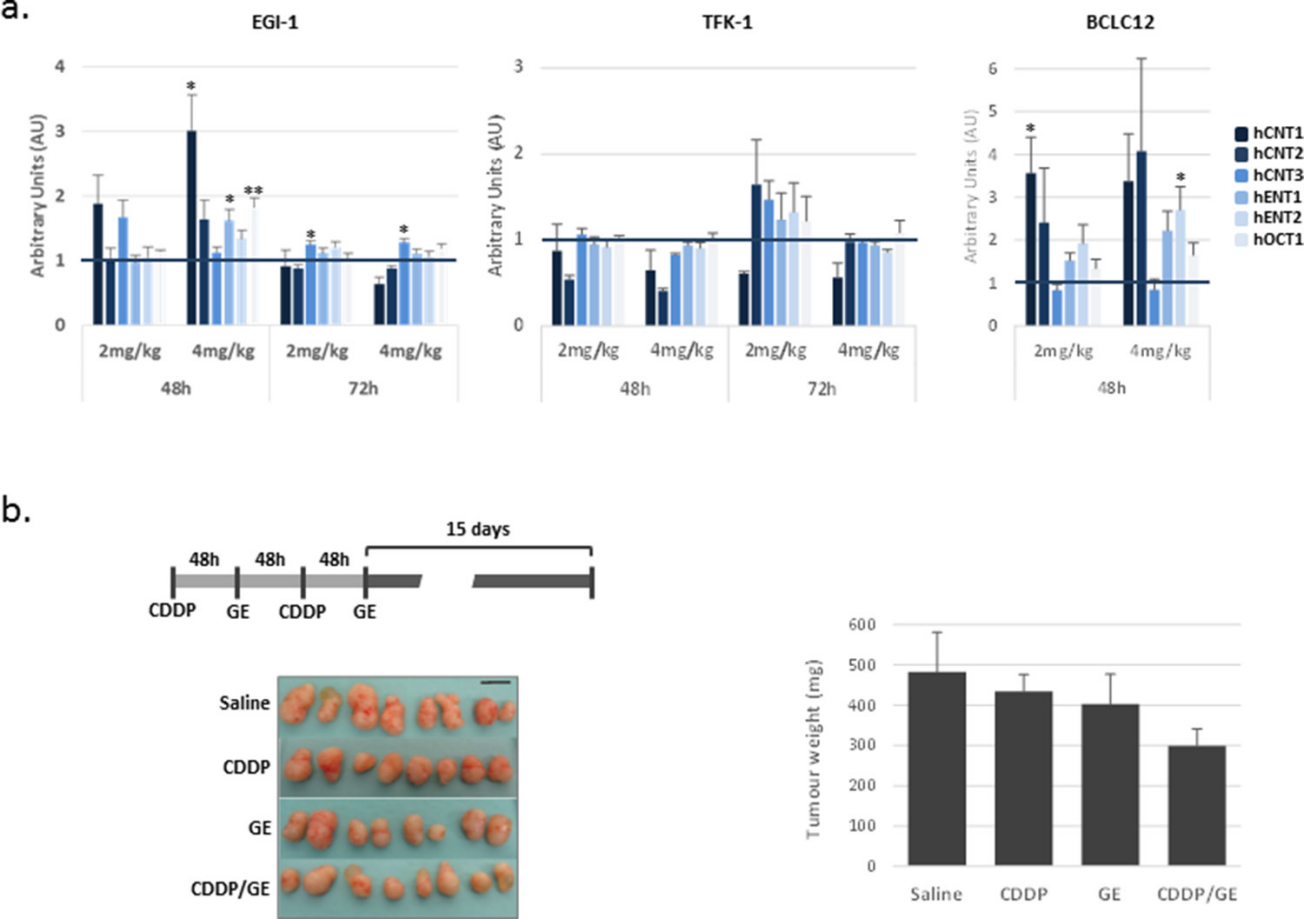
Delayed combination treatment potentiates gemcitabine effect in EGI-1 derived tumors **(A)** Effect of cisplatin treatment on drug transporters *in vivo*. Drug transporter expression after 48h and 72h of cisplatin treatment at 2 mg/kg and 4 mg/kg in EGI-1, TFK-1 and BCLC12 derived subcutaneous tumors. **(B)** Combination treatment of cisplatin and gemcitabine in EGI-1 derived tumors following the indicated treatment schedule. Tumor weight and tumor pictures at the end of the experiment. Bars are means ± S.E.M. (n=8). Statistical significance was determined with ANOVA.

The observations described above induced us to evaluate the expression of drug transporters after cisplatin treatment *in vivo*. To this end, we treated subcutaneous tumors established in nude mice by injection of EGI-1, TFK-1 and BCLC12 cell lines. When tumors reached a volume of 100mm^3^, mice were administered with 2 or 4 mg/kg of cisplatin and tumors were collected 48 and 72h later. BCLC12 derived tumors were only obtained at 48h due to the low engraftment ability of this cell line, reaching barely a 45% after 5 months. The analysis of the drug transporters expression pattern showed a similar profile to the *in vitro* assays. EGI-1 and BCLC12 tumors exhibited an increase in almost all the drug transporters, being the best condition 48h after 4 mg/kg of CDDP administration, whereas no significant changes were detected in TFK-1 tumors at the assayed doses (Figure 6A).

To evaluate a delayed combination treatment, we selected EGI-1 tumors treated with 4 mg/kg of CDDP to combine with either gemcitabine or sorafenib 48h later. When tumors reached a volume of 70mm^3^ mice were administered with 4 mg/kg of cisplatin and 48h later received 100 mg/kg of gemcitabine or 50 mg/kg of sorafenib. Mice were subjected to two cycles of treatment with a 48h lag and tumor growth was followed up for two more weeks. Under these conditions, combination treatment provoked a 40% decrease of tumor growth, whereas single drugs only induced slight decreases of 10% and 16%, for CDDP and gemcitabine, respectively (Figure 6B). Surprisingly, two separated doses of sorafenib treatment alone increased tumor growth at this treatment temporal pattern (Supplementary Figure 8).

## DISCUSSION

Cholangiocarcinoma is an aggressive disease with poor clinical outcome, mainly due to the late diagnosis that limits the therapeutic options. Cisplatin and gemcitabine combination is the current therapy used in unresectable or metastatic cholangiocarcinoma. However, the effect on patients is limited and surgery remains the only potentially curative therapy, although with high rate of disease recurrence [19].

Nowadays, there is no discussion about the relevance of drug transporters in clinical outcome [20]. The analysis of drug transporters expression even the characterization of its polymorphisms is becoming a standard to predict drug efficacy and safety. Clinical evidence has demonstrated its relevance in pharmacokinetics and pharmacodynamics likewise in combination schedules where drug transporters have been involved in clinically relevant drug-drug interactions. Moreover, several studies demonstrated alterations in drug transporters encoding genes in several cancers such as diminished expression of hCNTs and hOCT1 in different tumors [14, 18, 21–23].

Cisplatin is a broad-spectrum anticancer platinum agent and is active against a wide range of solid tumors, including ovarian, testicular, bladder, colorectal, lung, and head and neck cancers [24]. Interestingly, the study of mechanisms of resistance in liver cancers showed that cisplatin treatment induces changes in several genes involved in the uptake and efflux of anticancer drugs [18]. Our analysis of cisplatin treatment in cholangiocarcinoma cell lines showed increased expression of several transporters involved in anticancer drug uptake, including hCNTs, hENTs and hOCT1. Furthermore, results exhibited a trend to increase the activity of these transporters concomitant with their expression especially in EGI-1 and BCLC12 cell lines, whereas no increase in transport activity was observed in TFK-1. These observations suggested that proper temporal pattern administration of drugs could improve the outcome of currently used chemotherapy combination of cisplatin and gemcitabine, likewise combination of cisplatin and sorafenib to benefit from hOCT1 increased uptake.

Gemcitabine is a deoxycytidine analogue drug widely used against several solid tumors, including pancreatic, lung, breast, bladder, head, neck, thyroid an ovarian cancers [25]. Nucleoside transporters are necessary to allow the uptake of nucleoside analogues into the cells. Specifically, hCNT1, hCNT3, hENT1 and hENT2 mediate the uptake of gemcitabine into cells [7, 26, 27]. hENT1 intratumoral expression and responsiveness to gemcitabine has shown positive correlation in several solid tumors, including cholangiocarcinoma [16, 28–31]. Nevertheless, no conclusive clinical studies have been performed, when dealing with the other gemcitabine transporters and treatment outputs. Moreover, hCNT1 expression shows a trend to diminish in some cancer types such as gynecologic tumors, breast cancer and pancreatic cancer [21, 22, 32], which clearly could impair chemosensitivity to gemcitabine. In this sense, the increase in gemcitabine transporters expression and activity induced by cisplatin treatment clearly benefit the combination treatment.

Sorafenib is a multikinase inhibitor approved for the treatment of hepatocellular carcinoma, renal cell carcinoma and advanced thyroid carcinoma [33]. hOCT1, hOATP1B1 and hOATP1B3 are the transporters involved in sorafenib uptake [12, 13, 34]. There are a few studies dealing with the effect of sorafenib in biliary cancer patients, although mainly without promising clinical results [35–38]. However, negative results could be explained by the lack of selection of specific subtypes of biliary cancers. In this sense, a pilot prospective study has recently shown a modest effect of sorafenib combined with best supportive care in patients with advanced and unresectable intrahepatic cholangiocarcinoma [39]. This kind of cholangiocarcinoma presents an overlapping molecular profile with hepatocellular carcinoma according to the published molecular signature by Sia et al [3]. A recent retrospective study shows that a reduced intratumoral hOCT1 mRNA expression might play a role as a prognostic biomarker in sorafenib–based hepatocarcinoma therapy, resulting in a worse survival [40]. However, when we face an increased expression of hOCT1, the activity of the transporter should be considered. In this sense, our results with TFK-1 showed no correlation between hOCT1 expression and activity as we observed for NTs expression and activity. Moreover, in spite of the results obtained *in vitro*, that showed a good synergy combining cisplatin and sorafenib in EGI-1 and BCLC12, the outcome of the *in vivo* experiment clearly showed that daily treatment pattern of sorafenib cannot be changed. Unexpectedly, treatment with only two doses of sorafenib delayed 96h instead of daily treatment induced tumor growth, discarding changes in the administration temporal pattern.

The key role of transporters assuring drug bioavailability, pointed us to design a chemosensitization strategy based on the enhancement of their activity. Thus, in cholangiocarcinoma cell lines, cisplatin treatment induced an increase in drug transporter expression that can enhance their transport activity. Under these conditions, combination treatments of cisplatin with either gemcitabine or sorafenib at the desired drug combination schedule induced synergy, enhancing the *in vitro* cytotoxic effect in both cases. In this sense, high throughput studies of CDDP resistance has revealed a complex scenario of cell signaling activation pathways triggered by this drug that could eventually explain the expression changes observed in drug transporters [41]. However, the lack of correlation between induced expression changes and activity in TFK-1 cells highlights the complexity of alterations induced by CDDP treatment, emphasizing the need to go deeply in transportome knowledge to find the best option to anticipate drug bioavailability and action.

Whether this type of combined effects is tumor-specific or may show broader impact on cancer treatment is something else that should be further studied. Moreover this study should be also extended to other combined therapies in which one particular agent may impact on the bioavailability of another one in combined therapies. In this regard we recently showed that FLT3 inhibitors used in the treatment of acute pediatric leukemia can impact on particular drug transporter expression (i.e. hENT1), thereby affecting cytarabine action [42].

Essentially our data strongly support the concept that a proper temporal administration pattern can either increase or diminish the uptake of a second drug, taking profit in some cases of alterations induced by the previous treatment. In this sense, direct alterations in the transportome activity profile induced by drug treatment might be taken into account for the prediction of treatment outcome and should not be ignored when patients face drug combination schedules.

## MATERIALS AND METHODS

### Reagents

Cisplatin, uridine, 1-methyl-4-phenylpyridinium iodide (MPP), 4-nitrobenzyl-6-thioinosine (NBTI), dipyridamole 1,1’-diethyl-2,2’-cyanine iodide (d22) and quinidine were obtained from Sigma-Aldrich (USA). Sorafenib tosylate and gemcitabine hydrochloride were purchased from MedChem express (Sweden). [5,6-^3^H]-uridine and [Methyl-^3^H]-N-methyl-4-phenylpyridinium iodide ([^3^H]MPP^+^) were purchased from Campro Scientific (Germany).

### Cell lines

EGI-1 and TFK-1 cell lines were obtained from DMSZ (Deutsche Sammlung von Mikroorganismen und Zellkulturen) culture collection. TFK-1 was maintained in RPMI 1640 (Lonza Group Ltd, Switzerland) medium and EGI-1 in Dulbecco's modified Eagle's medium (DMEM) (Lonza Group Ltd). Both cell media were supplemented with 10% fetal bovine serum (FBS), 1% L-glutamine 200 mM (Lonza Group Ltd), and 1% Pen/Strep 10000 U/mL (Lonza Group Ltd). Mz-Cha-1 and Mz-Cha-2 were gently provided by Dr. A. Knuth and were maintained in RPMI 1640 (Lonza Group Ltd), 1% GlutaMAX™-1 (Gibco, Thermo Fisher Scientific Inc., USA), 1% Sodium Pyruvate (Gibco, Thermo Fisher Scientific Inc.), 1% MEM non-essential aa (Lonza Group Ltd), 1% L-Gln (Lonza Group Ltd), 1% Pen/Strep 1000 U/ml (Lonza Group Ltd), 10% FBS heat inactivated. BCLC12 and BCLC7 were generated from a patient with an intrahepatic cholangiocarcinoma. Tissue was collected in the operating room immediately after tumor excision. Liver dissociation was performed following standard protocol [43] with modifications. Briefly, tumor tissues were mechanically disaggregated and digested with collagenase IV (Sigma-Aldrich, St. Louis, MO). Tissue homogenates were filtered and centrifuged using Ficoll high-resolution density gradients. Cells were cultured at 37°C, 21% O_2_, 5% CO_2_ culture conditions. Primary cultures were submitted to successive subculture. Those cell cultures that were successfully passaged were further purified by means of single cell culture to obtain a clonal line. Specific antibodies targeting cytokeratins 7, CD56 and MUC1 were used to characterize cholangiocarcinoma cell line by means of immunocytochemistry. Standard culture medium for BCLC12 and BCLC7 cell lines is DMEM and F12 (1:1) (Lonza Group Ltd). Medium was supplemented with: 1% sodium pyruvate 100 mM (Gibco, Thermo Fisher Scientific Inc.), 1% Pen/Strep 10000 U/mL (Lonza Group Ltd), 1% Non-Essential Aminoacids (NEAA) (Lonza Group Ltd), and 10% FBS (Life Technologies, USA). Cell lines were maintained in proliferative conditions at 37 °C in a humidified atmosphere and a 5% CO_2_. All cell lines were confirmed to be mycoplasma free every two weeks by PCR amplification.

### RNA isolation and RT-PCR

Total RNA was isolated from cell lines and tumors using the SV Total RNA Isolation System (Promega, USA). A total of 1 μg of RNA was reverse transcribed to cDNA following M-MLV Reverse Transcriptase (Invitrogen, USA) and random hexamers (Amersham Pharmacia, UK) for reverse transcription. Analysis of hCNT1, hCNT2, hCNT3, hENT1, hENT2 and GAPDH (internal control) mRNA levels were performed by RT-PCR using TaqMan Gene Expression Assays (Applied Biosystems, USA) as previously described [44]. The mRNA expression of hOCT1 was assessed using the commercial Gene Expression Assays (Applied Biosystems). Relative quantification of gene expression was assessed using the ΔΔCT method, as described in the TaqMan user's manual (User Bulletin no. 2; Applied Biosystems). Gene expression levels for each individual sample were normalized relative to the GAPDH gene. The amounts of mRNA were expressed as arbitrary units.

Absolute quantification of gene expression was performed by using DNA plasmids containing each of the analyzed transporters to construct standard curves based on serial dilutions of the plasmids. The standard curves allowed us to correlate CT values of the samples with the mRNA copy number of each gene per microgram of total RNA.

### Transport assays

Nucleoside uptake was measured as described previously [45] by exposing replicate cultures at room temperature to [^3^H] labeled uridine (1 *μ*M, 1 *μ*Ci/ml) in sodium-containing or sodium-free transport buffer (137 mM NaCl or 137 mM choline chloride, 5 mM KCl, 2 mM CaCl_2_, 1 mM MgSO_4_, and 10 mM HEPES, pH 7.4). Initial rates of transport were determined using an incubation period of 1 min. Transport was stopped by washing with an excess volume of cold stop solution (173 mM choline chloride, 10 mM HEPES pH 7.4). hENT1 and hENT2 transport was discriminated inhibiting with 1 μM NBTI for hENT1 and 10 μM dipyridamole for both hENT1 and hENT2. [^3^H]MPP^+^ uptake rates mediated by hOCTs were measured in sodium-containing transport buffer. For hOCTs transport measurements, d22 OCTs inhibitor and quinidine OCT1 inhibitor were used.

Cells were then lysed in 100 *μ*l of 100 mM NaOH/0.5% Triton X-100. Aliquots were used for radioactivity counting and protein determination using the BCA reaction (Pierce, USA).

### Dose-response assays

Dose-response assays were performed exposing cells for 24 hours to cisplatin IC20 dose and 48 or 72 hours later cells were treated with increasing concentrations of gemcitabine or sorafenib for 24 hours. To avoid cell over-confluence, for 48 hours experiments, 2 × 10^5^ cells were seeded in 60 mm diameter culture plates 24 hours before treating cells with cisplatin, and for 72 hours experiments 1.5 × 10^5^ cells were seeded 72 hours before treating cells with cisplatin. 24 hours after cisplatin treatment, cell culture media was changed, and 5×10^3^ cells/well were seeded in 96-well culture plates. 72 hours after removing gemcitabine or sorafenib, cell viability was determined by MTT (3-[4,5-dimethylthiazol-2-yl]-2,5 diphenyl tetrazolium bromide) assay (Sigma-Aldrich).

Data were fitted to a dose–response curve using GraphPad Prism 4.0 software (GraphPad Software Inc., USA) to obtain 50% inhibitory concentration (IC50) values. Cell survival for all experiments was expressed as the percentage of viable cells relative to that in untreated cells (defined as 100%).

The coefficient of drug interaction (CDI) was used to analyze the effect of drug combination. CDI was calculated based on the absorbance in each group, as CDI = AB/(A × B), where AB is the ratio for the combination group relative to the control group, and A and B are the ratios of each single agent group relative to the control group. Thus, a CDI value < 1 indicates synergy, a CDI value = 1 indicates additive effects, and a CDI value > 1 indicates antagonism. CDIs less than 0.7 indicate a significant synergistic effect.

### Tumor growth studies

Tumor xenografts were developed by subcutaneous injection of 2 × 10^6^ TFK-1 cells, 6 × 10^6^ BCLC12 cells or 4 × 10^6^ EGI-1 cells into each posterior flank of female outbred nude mice (Charles River France, France). Tumor volume was measured three times a week and was calculated according to the equation, V(mm^3^)=π/6 × W × L^2^, where L and W are length and width of the tumor, respectively. Once tumors reached 100 mm^3^, mice were randomized (n=6 per group) and were treated with an intraperitoneal injection of saline, 2 mg/kg of cisplatin or 4 mg/kg of cisplatin. Tumors were collected 48 and 72h after treatment.

In combination studies, tumor xenografts were developed with EGI-1 cells as previously mentioned. Once tumors reached 70mm^3^, mice were randomized (n=4 per group) and two cycles of intraperitoneal injection of 4 mg/kg of cisplatin and 48h later followed by intraperitoneal injection of 100 mg/kg of gemcitabine or by oral gabage of 50 mg/kg of sorafenib. Tumor growth was followed up for 15 days after the end of the second cycle.

All animal procedures met the guidelines of European Community Directive 86/609/EEC and were previously approved by the Local Ethical Committee.

### Statistical analysis

Results were statistically analyzed using excel Student *t* test for comparisons between two groups. Comparisons among more than two groups were performed with GraphPad program using one-way analysis of variance (ANOVA) with Tukey's post-hoc. Differences were considered significant when p<0.05.

## SUPPLEMENTARY MATERIALS FIGURES AND TABLES



## Abbreviations

(CCA): cholangiocarcinoma
(NT): nucleoside transporter
(CNT): concentrative nucleoside transporter
(ENT): equilibrative nucleoside transporter
(OCT): organic cation transporter
(OATP): organic anion transporting polypeptides
(CDDP): cisplatin
