# Supplementary material for: Role of drug-dependent transporter modulation on the chemosensitivity of cholangiocarcinoma
Source: Oncotarget. 2017 Oct 6;8(52):90185–96. doi: 10.18632/oncotarget.21624 (PMC5685741; doi:10.18632/oncotarget.21624)
Supplement: Supplementary file 1 [file oncotarget-08-90185-s001.pdf]

Role of drug-dependent transporter modulation on the chemosensitivity of cholangiocarcinoma

SUPPLEMENTARY MATERIALS

Dose-response assays

For cisplatin cytotoxic effect determination, dose–response assays were performed by seeding 5 × 10^3 cells/well in 96-well culture plates. Cultures were exposed to increasing concentrations of cisplatin for 24 hours, and 48 hours after drug removing cell viability was determined by MTT assay.

For dose-response curves combining cisplatin treatment for 24h and gemcitabine, 5x10^3 cells/well were seeded in 96-well culture plates. 24 hours after cisplatin IC20 treatment, cells were exposed to increasing concentrations of sorafenib for 24 hours. 72 hours after drug removal cell viability was determined by MTT assay.

For cisplatin and 5-fluorouracil or paclitaxel combination assays, experiments were performed as explained before for cisplatin and gemcitabine or sorafenib combination in methods.

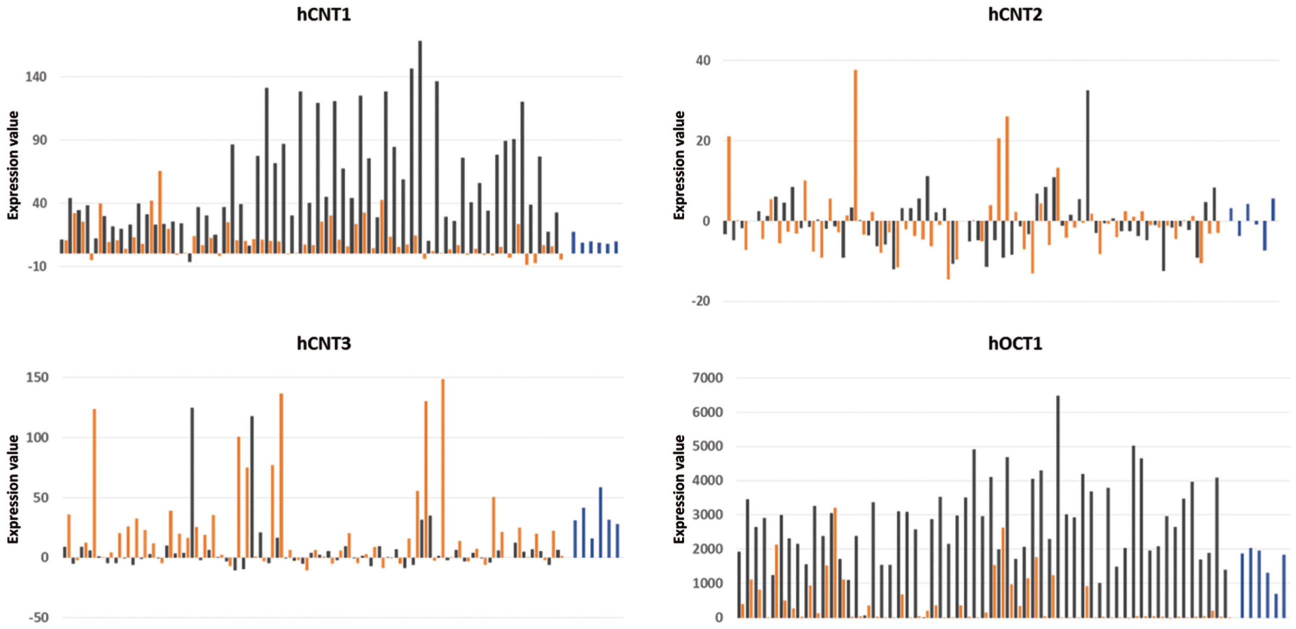

	hCNT1	hCNT2	hCNT3	hOCT1
Surrounding Liver	57.30 ± 5.42	-0.42 ± 0.94	6.73 ± 3.06	2763.7 ± 159.4
Cholangiocarcinoma tumor	11.11 ± 1.82	-0.40 ± 1.14	23.06 ± 4.94	439.4 ± 91.7
Normal Intrahepatic bile duct	10.38 ± 1.46	0.18 ± 2.06	34.51 ± 5.90	1620.0 ± 212.1
Paired student's t-test	<0.0001	0.99	0.007	<0.0001

Supplementary Figure 1: Cholangiocarcinoma drug transporters expression. **(A)** hCNT1, hCNT2, hCNT3 and hOCT1 expression was determined in matched surrounding livers and tumors from 59 CCA patients and 6 normal intrahepatic bile ducts using previously published data from GEO: GSE26566 [1]. Table results are expressed as mean ± S.E.M. Statistical significance was determined with Paired Student's t-test.

a.

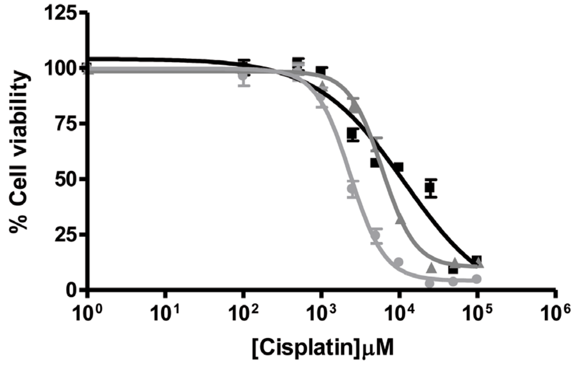

b.

Cell line	IC50 (μM)	IC20 (μM)
TFK-1	6.58 ± 0.70	3.18 ± 0.92
BCLC12	2.52 ± 0.42	1.25 ± 0.33
EGI-1	10.46 ± 3.27	2.53 ± 0.89

Supplementary Figure 2: Cisplatin cytotoxic effect determination. EGI-1, TFK-1 and BCLC12 cell lines were treated with increasing cisplatin doses for 24h. **(A)** Dose response-curves in EGI-1 (black), TFK-1 (gray) and BCLC12 (light gray). **(B)** IC50 and IC20 values.

a.

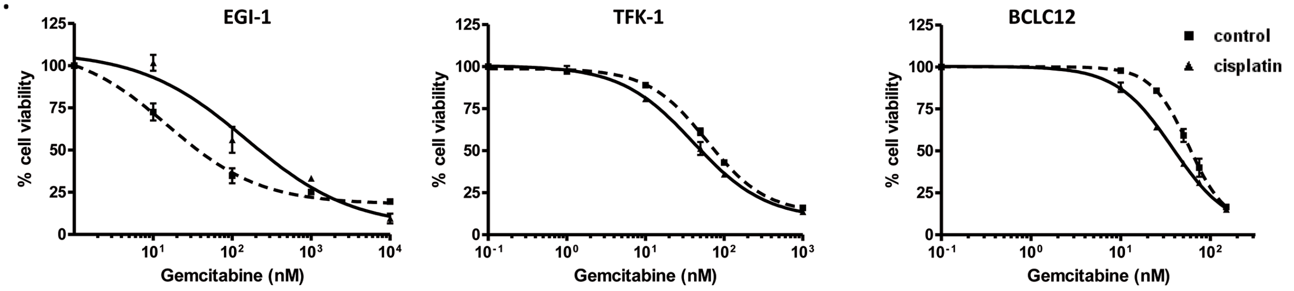

b.

[Gemcitabine] nM	CDI 0h
10000	0.3
1000	0.5
100	1.3
10	1.4

[Gemcitabine] nM	CDI 0h
1000	0.9
100	0.8
50	0.8
10	0.9
1	1.0

[Gemcitabine] nM	CDI 0h
150	0.9
75	0.8
50	0.7
25	0.7
5	0.9

Supplementary Figure 3: Dose-response curves combining gemcitabine and cisplatin treatment simultaneously. (A) EGI-1, TFK-1 and BCLC12 cell lines were treated at the same time with IC20 cisplatin dose (solid line) or vehicle (dashed line) and with gemcitabine increasing doses. **(B)** CDI values for CDDP and gemcitabine combination treatments at 0h. Results are expressed as mean ± S.E.M. (n=3).

a.

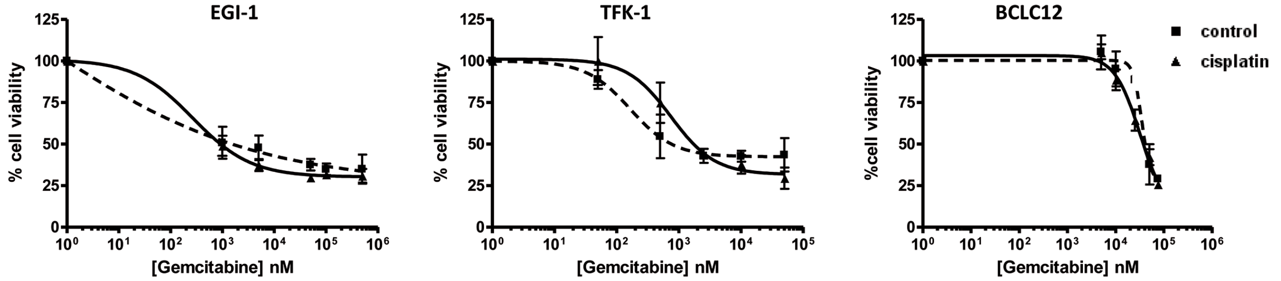

b.

[Gemcitabine] µM	CDI 24h
**500**	0.9
**100**	0.9
**50**	0.8
**5**	0.8
**1**	1.0

[Gemcitabine] µM	CDI 24h
**500**	0.7
**10**	0.9
**2.5**	1.0
**0.5**	1.4
**0.05**	1.1

[Gemcitabine] µM	CDI 24h
**75**	0.9
**50**	1.1
**25**	0.7
**10**	0.9
**5**	1.0

Supplementary Figure 4: Dose-response curves combining gemcitabine treatment after 24h of cisplatin. (A) EGI-1, TFK-1 and BCLC12 cell lines were treated with IC20 cisplatin dose (solid line) or vehicle (dashed line) and 24h later were treated with gemcitabine increasing doses. **(B)** CDI values for CDDP and gemcitabine combination treatments at 24h. Results are expressed as mean ± S.E.M. (n=3).

a.

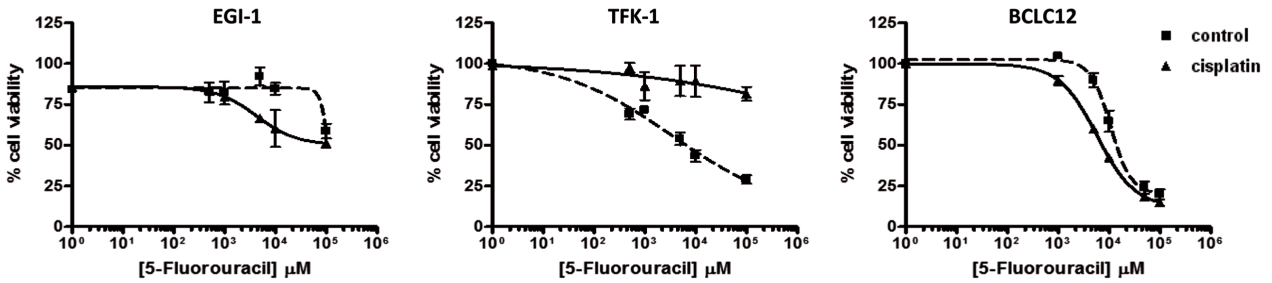

b.

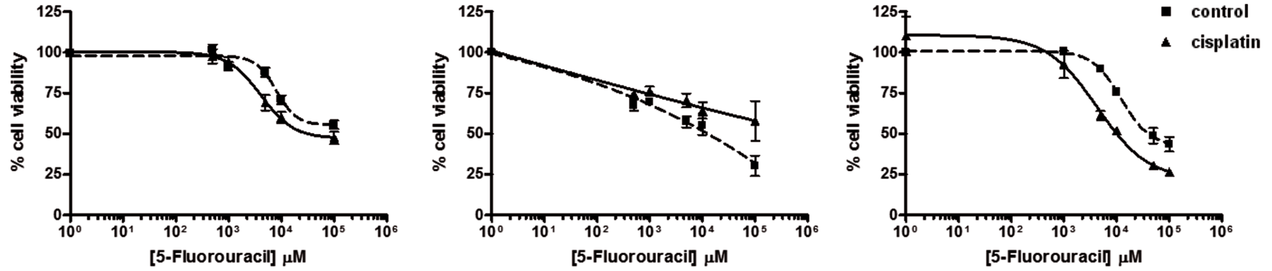

c.

[5-Fluorouracil] μM	CDI 48h	CDI 72h
**100**	0.9	0.3
**10**	0.7	0.5
**5**	0.7	0.5
**1**	0.9	0.5
**0.5**	1.0	0.5

[5-Fluorouracil] μM	CDI 48h	CDI 72h
**100**	2.8	1.9
**10**	2.1	1.2
**5**	1.7	1.2
**1**	1.2	1.1
**0.5**	1.4	1.1

[5-Fluorouracil] μM	CDI 48h	CDI 72h
**100**	2.8	1.9
**10**	2.1	1.2
**5**	1.7	1.2
**1**	1.2	1.1
**0.5**	1.4	1.1

Supplementary Figure 5: 5-Fluorouracil cytotoxic effect improves with the administration temporal pattern. Dose-response curves in EGI-1, TFK-1 and BCLC12 cell lines combining cisplatin and 5-fluorouracil. Cells were treated with IC20 cisplatin dose (solid line) or vehicle (dashed line) and 48h **(A)** and 72h **(B)** later were treated with 5-fluorouracil increasing doses. **(C)** CDI values for CDDP and 5-fluorouracil combination treatments at 48h and 72h. Results are expressed as mean ± S.E.M. (n=3).

a.

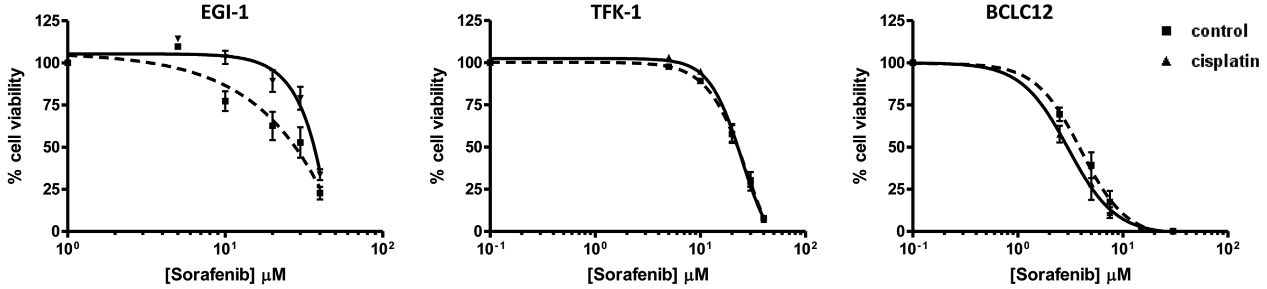

b.

[Sorafenib] µM	CDI 0h
40	1.5
30	1.5
20	1.4
10	1.3
5	1.0

[Sorafenib] µM	CDI 0h
40	0.9
30	1.0
20	1.0
10	1.1
5	1.1

[Sorafenib] µM	CDI 0h
30	2.2
15	1.4
7.5	0.6
5	0.7
2.5	0.8

Supplementary Figure 6: Dose-response curves combining sorafenib and cisplatin treatment simultaneously. (A) EGI-1, TFK-1 and BCLC12 cell lines were treated at the same time with IC20 cisplatin dose (solid line) or vehicle (dashed line) and with sorafenib increasing doses. **(B)** CDI values for CDDP and sorafenib combination treatments at 0h. Results are expressed as mean ± S.E.M. (n=3).

a.

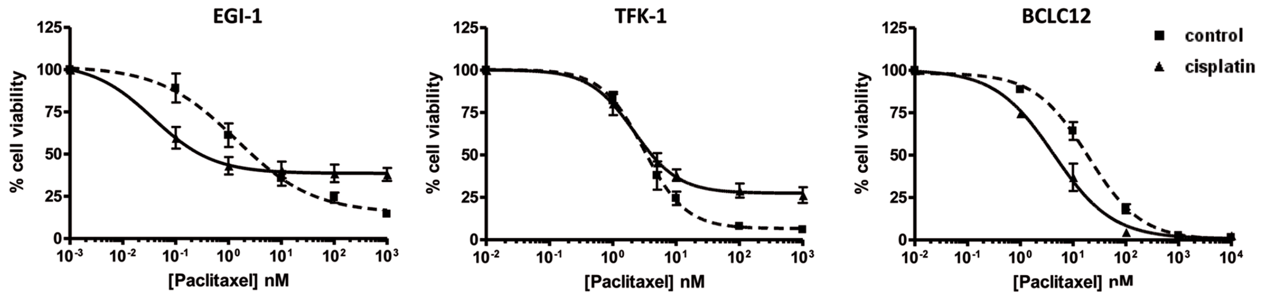

b.

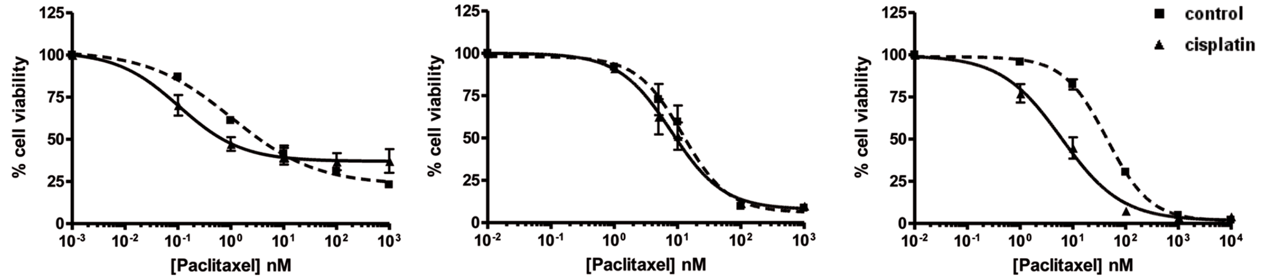

c.

[Paclitaxel] nM	CDI 48h	CDI 72h
1000	2.6	1.6
100	1.6	1.2
10	1.1	1.0
1	0.7	0.8
0.1	0.7	0.8

[Paclitaxel] nM	CDI 48h	CDI 72h
1000	4.4	1.2
100	3.6	1.2
10	1.6	0.9
5	1.2	0.9
1	1.0	1.0

[Paclitaxel] nM	CDI 48h	CDI 72h
10000	1.8	2.1
1000	1.0	0.8
100	0.3	0.2
10	0.6	0.5
1	0.8	0.8

Supplementary Figure 7: Paclitaxel cytotoxic effect improves with the administration temporal pattern. Dose-response curves in EGI-1, TFK-1 and BCLC12 cell lines combining cisplatin and paclitaxel. Cells were treated with IC20 cisplatin dose (solid line) or vehicle (dashed line) and 48h **(A)** and 72h **(B)** later were treated with paclitaxel increasing doses. **(C)** CDI values for CDDP and paclitaxel combination treatments at 48h and 72h. Results are expressed as mean ± S.E.M. (n=3).

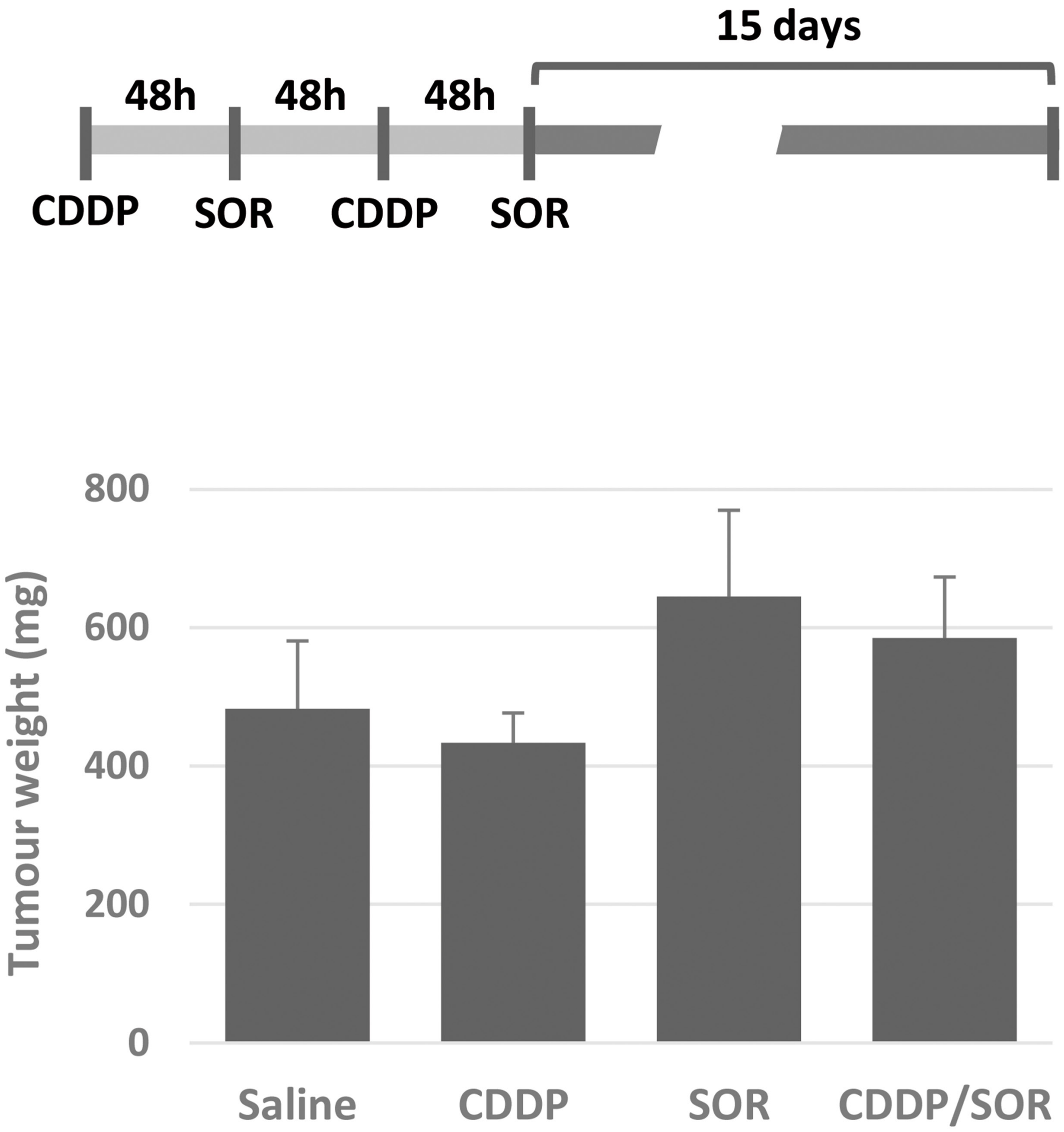

Supplementary Figure 8: Sorafenib treatment induces tumor growth in EGI-1 derived tumors. Combination treatment of cisplatin and sorafenib in EGI-1 derived tumors following the indicated treatment schedule. Tumor weight at the end of the experiment. Bars are means ± S.E.M. (n=8). Statistical significance was determined with ANOVA.

Supplementary Table 1: Characteristics of patients and tumors

	**Cholangiocarcinoma patients n=8**
Gender (M/F)	3/5
Age	68 ± 11.77
Tumor size	6,11 ± 2.69
Tumor differentiation degree good/poor/bad	2/3/3
Cirrhosis yes/no	2/6
